# Cell-Cell Connection Enhances Proliferation and Neuronal Differentiation of Rat Embryonic Neural Stem/Progenitor Cells

**DOI:** 10.3389/fncel.2017.00200

**Published:** 2017-07-21

**Authors:** Qian Jiao, Xingxing Li, Jing An, Zhichao Zhang, Xinlin Chen, Jing Tan, Pengbo Zhang, Haixia Lu, Yong Liu

**Affiliations:** ^1^Institute of Neurobiology, School of Basic Medical Sciences, Xi’an Jiaotong University Health Science Center Xi’an, China; ^2^Key Laboratory of Environment and Genes Related to Diseases, Ministry of Education of China, Xi’an Jiaotong University Beijing, China; ^3^Department of Physiology, Medical College of Qingdao University Qingdao, China; ^4^Department of Anesthesiology, The First Affiliated Hospital, Xi’an Jiaotong University Health Science Center Xi’an, China; ^5^Department of Anesthesiology, The Second Affiliated Hospital, Health Science Center, Xi’an Jiaotong University Xi’an, China

**Keywords:** NSCs/NPCs, cell-cell connection, proliferation, neuronal differentiation, MAPK

## Abstract

Cell-cell interaction as one of the niche signals plays an important role in the balance of stem cell quiescence and proliferation or differentiation. In order to address the effect and the possible mechanisms of cell-cell connection on neural stem/progenitor cells (NSCs/NPCs) proliferation and differentiation, upon passaging, NSCs/NPCs were either dissociated into single cell as usual (named Group I) or mechanically triturated into a mixture of single cell and small cell clusters containing direct cell-cell connections (named Group II). Then the biological behaviors including proliferation and differentiation of NSCs/NPCs were observed. Moreover, the expression of gap junction channel, neurotrophic factors and the phosphorylation status of MAPK signals were compared to investigate the possible mechanisms. Our results showed that, in comparison to the counterparts in Group I, NSCs/NPCs in Group II survived well with preferable neuronal differentiation. In coincidence with this, the expression of connexin 45 (Cx45), as well as brain derived neurotrophic factor (BDNF) and neurotrophin 3 (NT-3) in Group II were significantly higher than those in Group I. Phosphorylation of ERK1/2 and JNK2 were significantly upregulated in Group II too, while no change was found about p38. Furthermore, the differences of NSCs/NPCs biological behaviors between Group I and II completely disappeared when ERK and JNK phosphorylation were inhibited. These results indicated that cell-cell connection in Group II enhanced NSCs/NPCs survival, proliferation and neuronal differentiation through upregulating the expression of gap junction and neurotrophic factors. MAPK signals- ERK and JNK might contribute to the enhancement. Efforts for maintaining the direct cell-cell connection are worth making to provide more favorable niches for NSCs/NPCs survival, proliferation and neuronal differentiation.

## Introduction

The neurobiological behaviors of neural stem/progenitor cells (NSCs/NPCs), such as dormancy, proliferation or differentiation are affected by the environment where they are located (Bjornsson et al., 2015; Reinhard et al., 2016). In the neurogenic sites, niche signals that comprise cellular structures, extracellular matrix proteins and soluble factors drive the proliferation and differentiation of NSCs/NPCs (Ma et al., 2008; Solozobova et al., 2012). A better understanding of the signals that regulate NSCs/NPCs biological behaviors is clearly important. However, many studies on NSC biology were conducted and well discussed on the adult CNS and the specific niche signals for maintaining NSCs/NPCs (Kuo et al., 2006; He et al., 2015; Reinhard et al., 2016). By contrast, the niches for embryonic NSCs/NPCs had not been fully investigated.

Existed data had proved that NSCs/NPCs isolated from the developing brain tend to aggregate *in vitro* and easy to expand in 3-dimension (3-D) culture system (Justice et al., 2009). Svendsen has reported a method for rapid and long term growth of human NSCs/NPCs *in vitro* via maintaining the cell-cell contacts within the spheroids (Svendsen et al., 1998). Loss of the 3-D specific niche signals, growing on flat and hard glass or plastic substrates leads to the dramatic change of NSCs/NPCs behaviors (Pampaloni et al., 2007; Saha et al., 2008; Justice et al., 2009). Taking all these into account, it indicated the spatial relationship between NSCs/NPCs and their neighbor cells are critical for cell *in vitro* growth. Unfortunately, in the previous *in vitro* study approaches, the interactions of cells with one another and with the resulting extracellular microenvironments changes had not been properly addressed (Solozobova et al., 2012).

Recently, exploring the particular environmental cues for NSCs/NPCs proliferation and differentiation has become a major focus of research. Many factors, including mechanical and biochemical factors, and their effects on NSCs/NPCs fate decision have been explored. Gap junctions, as a mechanical cell-cell connection, as well as the small molecules that could pass through gap junction are essential for cell proliferation, migration and differentiation during brain development (Cheng et al., 2004; Elias et al., 2007; Khodosevich et al., 2012; Chapman et al., 2013; Naus et al., 2016). Biochemical cues, such as growth factors, neurotrophins, cytokines, neurotransmitters, etc., also paly critical roles in regulating NSCs/NPCs behaviors (Lathia et al., 2007; Sofroniew and Vinters, 2010). These factors could be produced by NSCs/NPCs, or the differentiated neurons, astrocytes, oligodendrocytes. In addition, MAPK signaling pathways are involved in the regulation of cell proliferation, survival, differentiation in the embryonic development and neurodegenerative disease (Miloso et al., 2008; Akchiche et al., 2010; Yoo et al., 2011). However, the details of how these factors works together to regulate NSCs/NPCs biological behavior still remain to be evaluated.

In the current study, the effects of cell-cell direct connection on rat embryonic NSCs/NPCs’ biological behaviors were investigated. Upon passaging, NSCs/NPCs spheres were either dissociated into single cell as usual (named Group I) or mechanically triturated into small cell clusters with the maintain of direct cell-cell connection (named Group II). Then the gap-junction between NSCs/NPCs and neurotrophic factors produced by NSCs/NPCs were addressed. The phosphorylation status of MAPK signals was also detected to uncover the underlying mechanisms.

## Materials and Methods

### Isolation and Culture of NSCs/NPCs

Pregnant female Sprague-Dawley rats were provided by Experimental Animal Center, Xi’an Jiaotong University Health Science Center. All procedures involving animal work conformed to the ethical guidelines of the NIH Regulations for Experimentation on Laboratory Animals and set out by the Xi’an Jiaotong University. The protocol was approved by the Institute of Neurobiology of Xi’an Jiaotong University.

NSCs/NPCs were isolated from cerebral cortex of rat embryos on embryonic day 14 (E14) to 15 (E15) and cultured in serum-free growth medium following the protocol of Gage et al. (1995) and optimized in our lab (Lu et al., 2011, 2013). NSCs/NPCs growth medium contains DMEM/F12 (Dulbecco’s modified Eagle medium and Ham’s F12, 1:1), 10 ng/mL bFGF, 20 ng/mL EGF, 100 U/mL penicillin, 100 μg/mL streptomycin, 1% N2 and 2% B27 supplement (all from Invitrogen, Carlsbad, CA, USA) and 2.5 μg/mL heparin (Sigma, St. Louis, MO, USA). The differentiation medium contains DMEM/F12 (1:1), 1% N2, 2% B27 supplement, 100 U/mL penicillin, 100 μg/mL streptomycin and 1% fetal bovine serum (FBS, Invitrogen, Carlsbad, CA, USA). Primary cultured cells were subcultured every 5 days.

### Experimental Design

NSCs/NPCs on passage number 2–3 were selected for current experiment. Cell aggregates were collected on the 5th day *in vitro* (DIV) and re-suspended by 2 mL medium in one test tube, and then the cell suspension was divided equally into two parts. One of them was centrifuged and suspended again with 100 μL medium, followed by trypsinization and mechanically trituration to make single cell suspension (Group I). The other part which contained 1 mL medium and cell aggregates were mechanically pipetted for 25–30 times to make a mixture of single cell and cell clusters (Group II). In Group II, the cell-cell connection was maintained within the clusters (Figure 2A). Then the NSCs/NPCs in both groups were cultured either in growth medium or differentiation medium following the standard protocols. The biological behaviors, in terms of survival, proliferation, differentiation were investigated.

**Figure 1:**
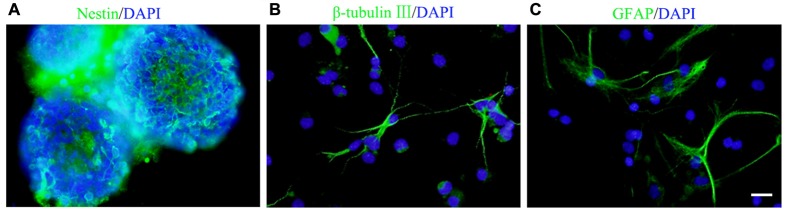
Culture and identification of neural stem/progenitor cells (NSCs/NPCs). **(A)** Most of the cells in spheres were nestin positive (5 DIV). **(B,C)** β-tubulin III positive neurons **(B)** and GFAP positive astrocytes **(C)** were observed after 7 days culture in differentiation medium. DIV, days *in vitro*. Scale bar = 20 μm. Nestin, β-tubulin III and GFAP were in green and DAPI was in blue.

**Figure 2:**
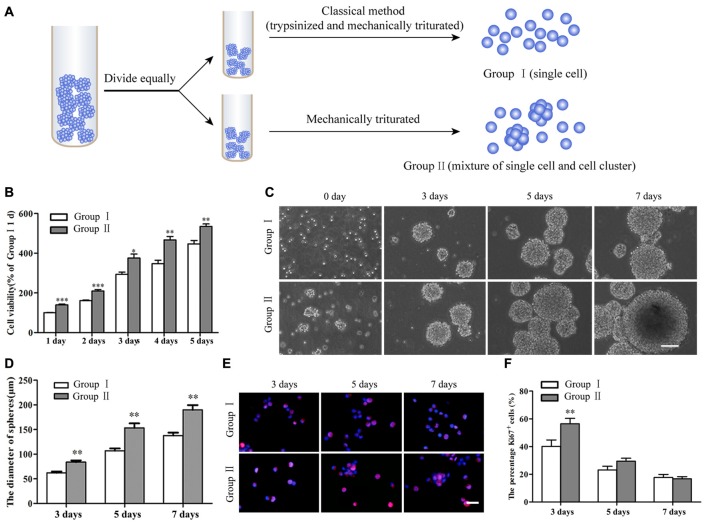
Survival and proliferation of NSCs/NPCs. **(A)** Sketch map of different subculture methods. **(B)** Cell counting kit-8 (CCK-8) result showed the better survival of NSCs/NPCs in Group II. **(C)** Different sizes of neurospheres formed along the culture. **(D)** Faster growth of NSCs/NPCs in Group II showed by the quicker increase of neurospheres’ diameter. **(E,F)** The expression of Ki67 in NSCs/NPCs in both groups. Scale bar = 100 μm **(B)** or 20 μm **(D)**. All data were obtained from three independent experiments and five replicates for every experiment. The values are mean ± SD and analyzed via Student’s *T* test. ^*^P < 0.05, ^**^P < 0.01, ^***^P < 0.001.

Gap junction communication is one type of cell-cell connection that occurs through intercellular channels formed by connexins. Hence, the expression of gap junction channel connexin 45 (Cx45) in NSCs/NPCs was investigated. In addition, the neurotrophic factors, including brain derived neurotrophic factor (BDNF) and neurotrophin 3 (NT-3), within the culture medium in both groups were compared. To further explore the possible molecular mechanisms, the phosphorylation status of MAPK signals, including ERK1/2, JNK2 and p38 were detected and then the NSCs/NPCs biological behaviors were re-analyzed after applying the antagonists, including U0126 (a highly selective inhibitor of ERK), SP600125 (broad-spectrum JNK inhibitor) and SB202190 (a selective inhibitor of p38), respectively. All antagonists (all from Sigma, St. Louis, MO, USA) were dissolved in DMSO and then diluted with medium to make a solution with different concentration (1 μM, 5 μM and 10 μM). All of above assays were triplicated.

### Cell Viability and Proliferation Assay

NSCs/NPCs in both groups were cultured in growth medium and cell counting kit-8 (CCK-8, Dojindo, Peking, CN) was applied to analyze cell viability following the standard protocol. Equal numbers of NSCs/NPCs (about 10,000) were suspended in 100 μL growth medium and added into each well of 96-well plates. A water-soluble tetrazolium salt (WST-8, [2-(2-methoxy-4-nitrophenyl)-3-(4-nitrophenyl)-5-(2,4-disulfophenyl)-2H-tetrazolium], 10 μL) was added into each well and then followed with for 2–4 h incubation at 37°C. WST-8 will be reduced by dehydrogenases in cells to give an orange colored product (formazan). The amount of the formazan dye is directly proportional to the number of living cells. Based on this, cells were observed every day for 5 days by using an UV-1750 spectrometer reader (Shimadzu, JP) at 450 nm. The OD value of Group I at day 1 was considered as 100%.

In addition, NSCs/NPCs from two groups were also cultured in 6-well plated and the diameters of 20 neurospheres in each group were measured at different time points to illustrate the growth of NSCs/NPCs. Then at 7 DIV, all neurospheres in both groups were collected and trypsinized into single cell, the number of NSCs/NPCs was counted to further confirm the growth of NSCs/NPCs. NSCs/NPCs from two groups were also cultured in 24-well plate in growth medium and the proliferation of NSCs/NPCs was assessed by Ki67 expression assay at 3, 5 and 7 DIV, respectively. The percentage of Ki67^+^ cells out of total cells was calculated.

### Immunocytochemistry Staining

Immunocytochemistry staining was performed following standard protocol and optimized in author’s laboratory (Lu et al., 2011, 2013). Primary antibodies, including monoclonal mouse anti-nestin (1:200, Millipore, Temecula, CA, USA), monoclonal mouse anti-β-tubulin III (1:200, Millipore, Temecula, CA, USA) and monoclonal mouse anti-GFAP (1:500, Millipore, Temecula, CA, USA) were used to identify NSCs/NPCs, neurons, astrocytes, respectively. Monoclonal rabbit anti-Ki67 (1:300, Millipore, Temecula, CA, USA) was used to identify the proliferating cell. All primary antibodies used in this study were diluted in PBS with 2% normal goat serum (NGS). Blocking solution contained 5% NGS and 0.25% Triton X-100 in PBS. TRITC- and FITC-conjugated goat anti-mouse IgG or anti-rabbit IgG (1:400; CWBIO, Peking, CN) were used as secondary antibodies. Cell nuclei were counterstained with DAPI-containing mounting media (Vector, Burlingame, CA, USA) and visualized under a fluorescent microscope (Olympus BX57) equipped with a DP70 digital camera and the DPManager (DPController, Olympus) software. For the negative control, primary antibody was replaced by blocking buffer.

For NSCs/NPCs identification, cell aggregates (for nestin) or single cells (for β-tubulin III and GFAP) were seeded onto poly-L-lysine (PLL) coated coverslips. For the immunostaining of Ki67, cell aggregates from both groups were dissociated into single cells and “re-seeded” onto the PLL coated coverslips before staining. For NSCs/NPCs differentiation assessment, cells from both groups were cultured in differentiation medium on coverslips for 7 days. Before staining, all cells were fixed with 4% paraformaldehyde (PFA) for 20 min at room temperature.

### Quantitative Real-Time PCR and ELISA Assay

NSCs/NPCs in both groups were cultured in 6-well plates either in growth medium or differentiation medium. After 1, 2 and 3 days culture, total RNA of NSCs/NPCs were extracted for the investigation of Cx45 expression and the culture medium was collected for the detection of neurotrophic factors.

Cx45 expression was detected by quantitative Real-time PCR (qRT-PCR). Total RNA was extracted by using the Trizol reagent (Invitrogen) in accordance with manufacturer’s protocol and was quantified by spectrophotometry (Nano-Drop^™^ Wilmington, DE, USA). RNAs were reversely transcribed to cDNA by a reverse transcriptase kit (PrimeScript^™^ RT reagent Kit, TaKaRa, Dalian, CN). Relative abundance of each mRNA was quantified by qRT-PCR using specific primers and the SYBR® Premix Ex TaqII (TaKaRa, Dalian, CN). Primers for rat Cx45 and GAPDH were (forward 5′-AAAGAGCAGAGCCAACCA-3′; reverse 5′-GAATGGTCCCAAACCCTAGAT-3′) and (forward 5′-ACCACAGTCCATGCCATCAC-3′; reverse 5′-TCCACCACCCTGTTGCTGTA-3′), respectively (Talaverón et al., 2015). QRT-PCR reactions were carried out by using iQ^™^ Multicolor Real-Time PCR Detection System (BIO. RAD, USA). Cycle threshold values were obtained from the BIO-RAD iQ5 2.0 Standard Edition Optical System Software (BIO. RAD, USA). Data were analyzed by the ^∆∆^CT method, and GAPDH was taken as an internal control. The mRNA level of Cx45 in Group I at day 1 was considered as 1-fold.

Secretion of neurotrophic factors was detected by ELISA (Ray Biotech, GA, USA) assay. Medium were collected from three individual wells. Following the instruction, a volume of 50 μL medium was added to appropriate well and then incubated with 100 μL HRP-conjugated reagents at 37°C for 60 min. After thoroughly wash, chromogen solutions were added and incubate at 37°C. The reaction was stopped with stop solution 15 min later and the result was read at 450 nm in an EL808 Ultra Microtiter plate Reader (BIO-TEK instruments, VT, USA).

### Western Blot Analysis

The expression of nestin, β-tubulin III and GFAP were quantified by Western blot assay to further investigate the differentiation of NSCs/NPCs. The expression of MAPK signals, including ERK1/2 and p-ERK 1/2, JNK2 and p-JNK2, p38 and p-p38 were detected to identify the potential mechanisms of cell behavior changes. Therefore, besides the above monoclonal mouse anti-nestin (1:1000), anti-β-tubulin III (1:1000) and anti-GFAP (1:1000) antibodies, following primary antibodies, including monoclonal rabbit anti-ERK1/2 (1:2000) and anti-p-ERK 1/2 (1:2000), polyclonal rabbit anti-JNK2 (1:1000) and anti-p-JNK2 (1:2000), polyclonal rabbit anti-p38 (1:2000) and polyclonal rabbit anti-p-p38 (1:1000; All these from Cell Signaling Technology, Danvers, MA, USA), polyclonal rabbit anti-Cx45 (1:1000, Millipore, Temecula, CA, USA), monoclonal mouse anti-tubulin (1:10000, Abcam, Cambridge, UK), and monoclonal mouse anti-β-actin (1:5000, Santa Cruz Bio, Santa Cruz, CA, USA) were used. NSCs/NPCs were lysed in RIPA lysis buffer. Insoluble materials were removed by centrifugation at 12,000 rpm for 15 min at 4°C. Cell lysates were subjected to electrophoresis using 10% SDS polyacrylamide gels and transferred to nitrocellulose membranes. The membranes were incubated with primary antibodies at 4°C overnight and then with secondary antibodies at room temperature for 2 h after blocked in 5% non-fat dry milk in TBST for 2 h. Blots were detected by chemiluminescence with the ECL method (Pierce Biotechnology, Rockford, IL, USA) and the data were analyzed with ImageJ (version 1.61).

### Statistical Analysis

All data were analyzed with the SPSS 17.0 software. Student’s *T* test and one-way analysis of variance (ANOVA) were used. *P* value <0.05 was considered significant.

## Results

### Alteration of NSCs/NPCs Survival, Proliferation and Differentiation

Cells isolated from the rat embryonic cerebral cortex were cultured in DMEM/F12 serum free growth medium for 5 days, neurospheres in different sizes were observed. Immunocytochemistry staining showed that most of cells in neurospheres were nestin^+^ NSCs/NPCs (Figure 1A). After 7 days culture in differentiation medium, those cells could differentiate into β-tubulin III^+^ neurons and GFAP^+^ astrocytes (Figures 1B,C).

NSCs/NPCs in passage number 2 or 3 were subcultured through different ways as described above (Figure 2A). CCK-8 assay showed that there were more viable NSCs/NPCs in the Group II than that in Group I (Figure 2B). After several days culture, although neurospheres gradually formed in both groups (Figure 2C), neurospheres in Group II were significantly larger, indicating the quicker growth of NSCs/NPCs (Figure 2D). The numbers of NSCs/NPCs on 7th day were 1.20 ± 0.11 × 10^6^/well (Group I) and 2.01 ± 0.13 × 10^6^/well (Group II), respectively. It further confirmed the quicker cell growth in Group II. In accordance with this, significantly more Ki67^+^ proliferating cells were observed in Group II at the early stage of culture than that in Group I (Figures 2E,F).

**Figure 3:**
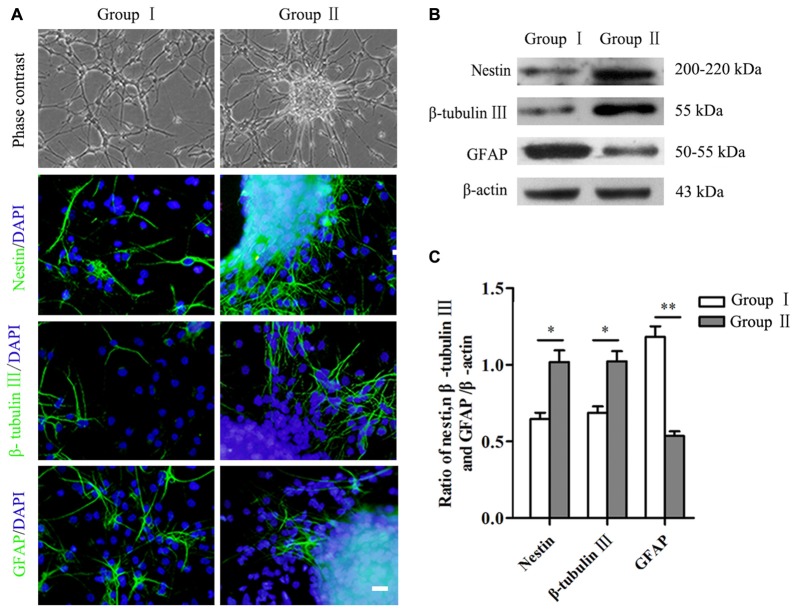
Differentiation of NSCs/NPCs. **(A)** Morphology and differentiation of NSCs/NPCs in both groups in differentiation medium (7 DIV), showed by immunostaining. **(B,C)** Significantly higher level of nestin, β-tubulin III and lower level of GFAP in Group II, showed by Western blot assay. Scale bar = 20 μm. The data were obtained from three independent experiments and three replicates for each experiment. The values are mean ± SD and analyzed via Student’s *T* test. Group II vs. Group I ^*^P < 0.05, ^**^P < 0.01.

Differentiation of NSCs/NPCs in both groups was detected by both immunocytochemistry staining and Western blot assay. After 7 days culture in differentiation medium, a large number of the cells still were nestin^+^ NSCs/NPCs in both groups, especially in Group II. In addition, in Group II, a significantly more cells undertook neuronal differentiation (Figure 3A). Quantification of proteins showed the same results. A remarkably higher level of nestin and β-tubulin III were detected in Group II than that in Group I, while the level of GFAP was opposite (Figures 3B,C, *P* < 0.05).

### Expression of Cx45 and Neurotrophic Factors in Different Groups

**Figure 4:**
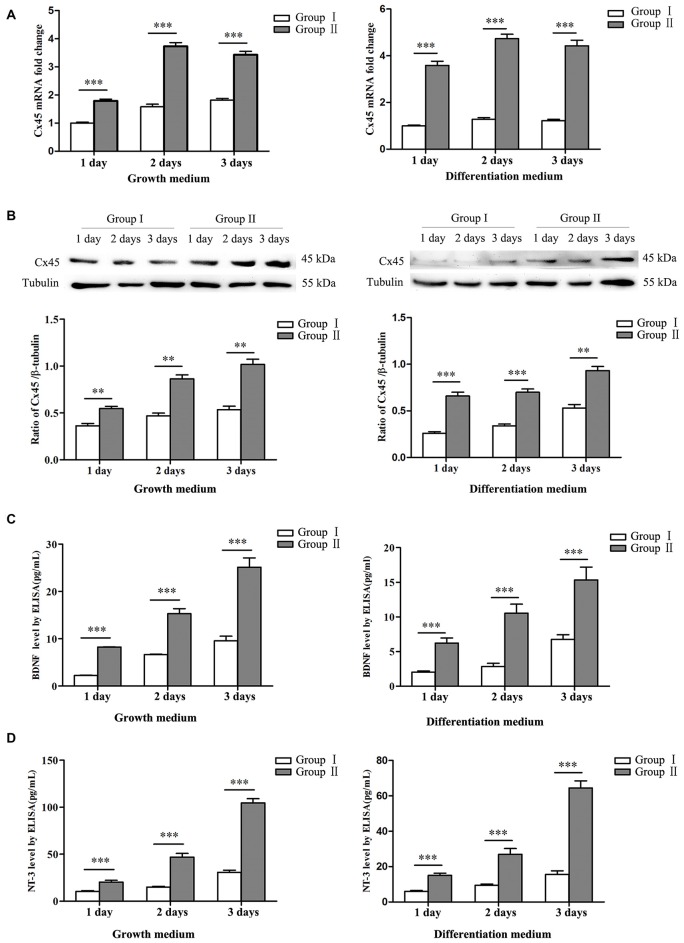
Expression of connexin 45 (Cx45), brain derived neurotrophic factor (BDNF) and neurotrophin 3 (NT-3). **(A)** Significantly higher mRNA levels of Cx45 in NSCs/NPCs in the Group II were detected, both in growth medium (left) and differentiation medium (right). **(B)** Significantly higher protein levels of Cx45 in NSCs/NPCs in the Group II were detected, both in growth medium (left) and differentiation medium (right). **(C)** Significantly higher levels of BDNF was secreted from NSCs/NPCs in the Group II, detected by ELISA assay, both in growth medium (left) and differentiation medium (right). **(D)** Significantly higher levels of NT-3 was secreted from NSCs/NPCs in the Group II, detected by ELISA assay, both in growth medium (left) and differentiation medium (right). The data were obtained from three independent experiments and three replicates for every experiment. The values are mean ± SD and analyzed via Student’s *T* test. ^**^P < 0.01, ^***^P < 0.001.

NSCs/NPCs were cultured in both growth medium and differentiation medium. Expressions of Cx45 were detected by qRT-PCR and Western blot analysis. The result showed that expression of Cx45, both in mRNA level and protein, in Group II was significant higher than that in Group I. Similar variation pattern was observed in both growth medium and differentiation medium (Figures 4A,B, *P* < 0.001). At same time, the protein levels of BDNF and NT-3 in culture medium were detected by ELISA assay. Result showed that, in comparison with NSCs/NPCs in Group I, significantly more BDNF and NT-3 were produced by NSCs/NPCs in Group II. The variation tendency was also independent with the culture medium (Figures 4C,D, *P* < 0.001).

### Expression and Function Assessment of MAPK Signals in Different Groups

**Figure 5:**
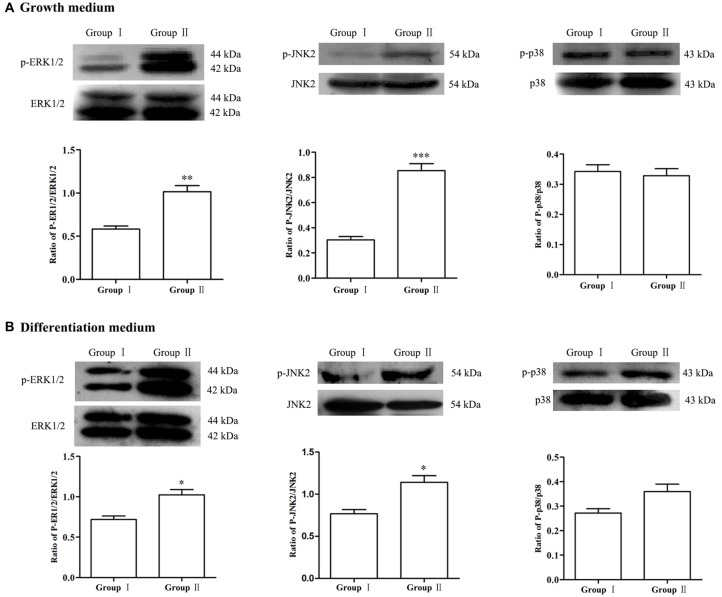
Alteration of MAPK signals. In comparison with Group I, the level of p-ERK1/2 and p-JNK2 significantly increased in the Group II while no significant difference was observed in regarding of p38, both in growth medium **(A)** and differentiation medium **(B)**. The data were obtained from three independent experiments and three replicates for every experiment. The values are mean ± SD and analyzed via Student’s *T* test. Group II vs. Group I ^*^P < 0.05, ^**^P < 0.01, ^***^P < 0.001.

To explore the possible molecular mechanisms, expression of MAPK signals was detected by Western blot analysis. Results showed that, in comparison with Group I, the phosphorylation of ERK and JNK in the NSCs/NPCs in Group II significantly increased, especially when NSCs/NPCs were cultured in growth medium (Figure 5, *P* < 0.05). However, no significant difference was observed in phosphorylation level of p38 (*P* > 0.05), either in growth medium or differentiation medium.

**Figure 6:**
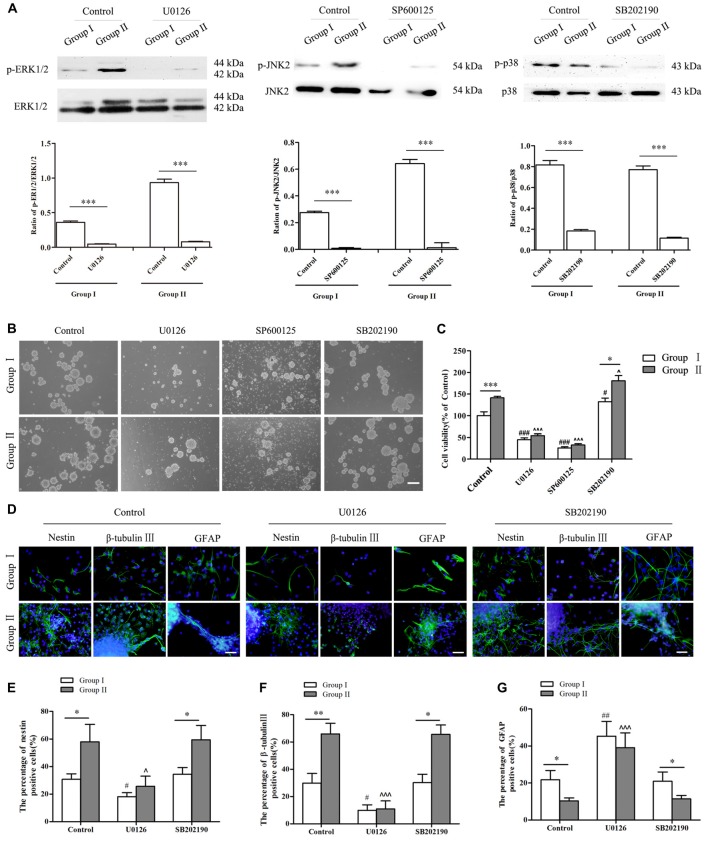
Alterations of NSCs/NPCs survival, proliferation and neuronal differentiation after the inhibition of MAPK signals. **(A)** The phosphorylation of ERK1/2, JNK2 and p38 significantly decreased after treated with U0126, SP600125 and SB202190 in 10 μM both in Group I and Group II. **(B)** Survival of NSCs/NPCs after U0126, SP600125 and SB202190 treatment for 2 days. Serious cell death was observed after the treatment of U0126 and SP600125 in both groups. **(C)** CCK-8 assay showed that U0126 and SP600125, not SB202190, treatment significantly reduced the survival and the viability of NSCs/NPCs in both groups. **(D–G)** Immunocytochemistry staining showed that the differentiation of NSCs/NPCs with treatment of U0126 and SB202190. The portion of nestin^+^ and β-tubulin III^+^ cells reduced significantly and the astrocytic differentiation of NSCs/NPCs was enhanced. They all were significantly different with control. No difference was found between control group and the treatment with SB202190. Scale bar = 100 μm **(B)** or 50 μm **(D)**. The data were obtained from three independent experiments. There were five replicates for every experiment in **(C)**, and three replicates for every experiment in** (A,E,F,G)**. The values are mean ± SD, analyzed via Student’s *T* test and one-way analysis of variance (ANOVA). ^*^P < 0.05, ^**^P < 0.01 and ^***^P < 0.001 (via Student’s *T* test); ^#^*P* < 0.05, ^##^*P* < 0.01 and ^###^*P* < 0.001 represent Group I vs. control (via one-way ANOVA); ^∧^*P* < 0.05 and ^∧∧∧^*P* < 0.001 represent Group II vs. control (via one-way ANOVA).

**Figure 7:**
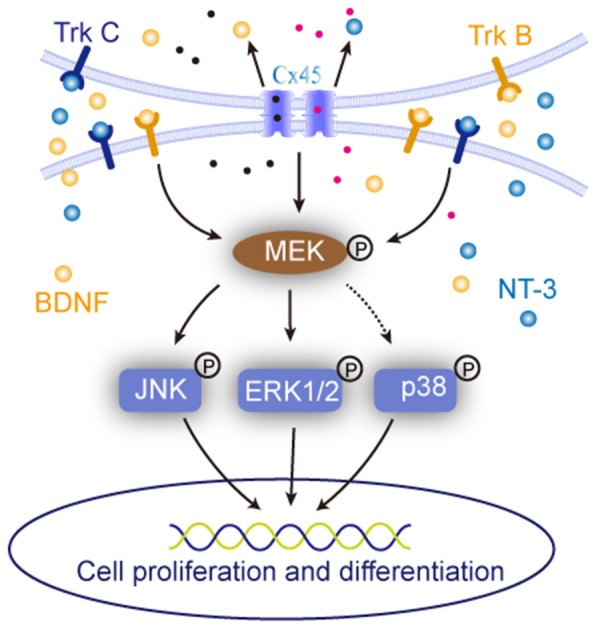
Sketch map of the roles of cell-cell interaction in regulating NSCs/NPCs behaviors. It suggested that when the cell-cell interaction was maintained, the expression of BDNF, NT-3 and were upregulated and their downstream MAPK signals responded to the alteration.

To further confirm the involvement of MAPK signals in regulating the biological behavior of NSCs/NPCs in different groups, antagonists including U0126 (ERK inhibitor), SP600125 (JNK inhibitor) and SB202190 (p38 inhibitor) were used. Lower concentrations (1 μM and 5 μM) of inhibitors showed no significant reduction of NSCs/NPCs viability in Group II. By coincidence, the phosphorylation of ERK and JNK did not meet any significant change (Supplementary Figures S1,S2, *P* > 0.05). However in Group I, NSCs/NPCs viability and phosphorylation of ERK and JNK were reduced significantly after treated with 5 μM of U0126 and SP600125 (Supplementary Figures S1, S2, *P* < 0.05). Contrarily, after 2 days incubation with 10 μM of U0126 and SP600125, the phosphorylation of ERK/JNK and NSCs/NPCs viability significantly decreased in both groups, particularly with the treatment of SP600125 (Figures 6A–C). Besides cell viability alteration, the neuronal differentiation of NSCs/NPCs reduced significantly and the astrocytic differentiation was promoted dramatically (Figures 6D–G, Supplementary Figure S3). It is notable that the differences of NSCs/NPCs survival and differentiation between two groups were disappeared when the phosphorylation of ERK and JNK reduced significantly. In coincidence with no difference of phosphorylation level of p38 between Group I and II, after the treatment with SB202190, the pattern of NSCs/NPCs biological behavior was maintained as similar as control. It suggested that ERK and JNK signals, but not p38, were involved and contributed to the enhancement of NSCs/NPCs survival and neuronal differentiation in Group II (Figure 7).

## Discussion

Cell-cell communication is critical for NSCs/NPCs survival, proliferation and differentiation (Solozobova et al., 2012; Ottone and Parrinello, 2015; Bosone et al., 2016). In the current study, rat embryonic NSCs/NPCs aggregates were passaged by two different ways, either been thoroughly dissociated into single cells or been mechanically pipetted into small cell clusters. A better survival and faster growth of NSCs/NPCs, higher percentage of neuronal differentiation accompanied with elevated levels of Cx45, BDNF and NT-3 were observed when the cell-cell connection was well maintained. MAPK signals, ERK and JNK, not p38, might be involved in the biological behavior changes of NSCs/NPCs.

Super advantages of 3-D culture system in maintaining stem cell characteristics have been deeply studied (Pampaloni et al., 2007; Choi et al., 2009; Justice et al., 2009; Lu et al., 2012). In spheroids, the spatial relationship and the intercellular niche are more favorable for neuronal precursors’ survival and differentiation than 2-D monolayer cultures (Lu et al., 2012). We noticed that NSCs/NPCs preferably aggregated together after passaging, especially in Group I. This indicated that NSCs/NPCs in culture tended to seek direct cell-cell connection and create an appropriate environment, spontaneously (Ladiwala et al., 2012). Consistently, in the current study, NSCs/NPCs survived better and proliferated faster in Group II where the cell-cell contacts were maintained. On the country to Group I, cell viability reduced significantly, particularly at the early stage when the cell-cell connection has not yet been established.

It has been proved that the proliferation rate of NSCs/NPCs was linear and depended on the size of the aggregate (Mori et al., 2006; Singec et al., 2006). In agreement with this, the percentage of Ki67^+^ cells in Group II was significantly higher than that in Group I before its cell-cell contacts had been set up. After few days culture, NSCs/NPCs in Group I aggregated together and created their own niches, then the difference of proliferation between two groups reduced.

Moreover, we noticed that significantly more NSCs/NPCs in Group II differentiated into β-tubulin III^+^ neurons, rather than GFAP^+^ astrocytes. This could be due to the appropriate microenvironment in Group II. NSCs/NPCs are tending to differentiate into neurons under a proper niche (Ming and Song, 2011; Keung et al., 2012), whereas to differentiate into astrocytes when exposure to harmful stimulation (Czéh et al., 2007; Encinas et al., 2011). It further confirmed that cell-cell connection is very much essential for NSCs/NPCs survival, proliferation and neuronal differentiation.

Connexins or gap junction proteins assemble to form one kind of cell-cell connection that essential for many physiological processes in neural development and neurogenesis (Elias et al., 2007, 2010; Kunze et al., 2009; Khodosevich et al., 2012; Clasadonte and Haydon, 2014). Cx45 is one kind of gap junction channels that is highly expressed during embryogenesis in nearly all brain regions (Maxeiner et al., 2003). It positively influenced neural progenitor cells and the expression level of Cx45 modulated the proliferation of transit-amplifying precursor cells (Khodosevich et al., 2012). In the current study, the expression of Cx45 in Group II was significantly elevated, and this might be the main reason for the better survival, faster proliferation and preferable neuronal differentiation of NSCs/NPCs.

Accompanied with the increase of Cx45, the levels of BDNF and NT-3 in the culture medium from Group II were significantly higher than that in Group I. These neurotrophic factors could be produced by NSCs/NPCs, or the differentiated cells. We know that neurotrophic factors are not only responsible for survival and growth of developing neurons and the function performance of mature neurons, but also play important roles in NSCs/NPCs proliferation and differentiation (Kamei et al., 2007; Yang et al., 2010; Zhang et al., 2011). Previously, we have demonstrated that NT-3 promoted human fetal NSCs survival, proliferation and neuronal differentiation *in vitro* (Lu et al., 2011). Here, the increased level of neurotrophic factors in Group II indicated their contribution to the promotion of NSCs/NPCs survival, proliferation and neuronal differentiation. Notably, similar with the expression of Cx45, the alteration of neurotrophic factors levels was independent with the culture medium. Further studies to figure out the intrinsic cues that trigger the expression of BDNF and NT-3 within those two different groups are still needed.

MAPK signaling pathways are involved in the regulation of proliferation, survival, differentiation and process extension in embryonic development and neurodegenerative disease (Akchiche et al., 2010; Yoo et al., 2011; Schröter et al., 2015). They are also the downstream signals of BDNF and NT-3 (Lim et al., 2007; Castillo and Escobar, 2011; Revest et al., 2014). In the current study, in order to further explore the possible molecular mechanisms for NSCs/NPCs biological alteration, the expression and phosphorylation status of MAPK signals, including ERK, JNK and p38, were observed. Corresponding to the changes of NSCs/NPCs biological behaviors, the phosphorylation of ERK and JNK, but not p38, in Group II was upregulated. When the ERK and JNK activation were blocked by their inhibitors, the differences of NSCs/NPCs proliferation and neuronal differentiation between two groups almost disappeared. Interestingly, lower concentration (5 μM) of inhibitors could significantly reduce the phosphorylation of ERK and JNK and change the behaviors of NSCs/NPCs in Group I, but not in Group II. It further confirmed the enhanced activation of ERK and JNK in Group II and this might contribute to the promotion of NSCs/NPCs survival and neuronal differentiation. Further investigation between the ERK/JNK activation and cell-cell connection is required.

In conclusion, direct cell-cell connection played important roles in regulating NSCs/NPCs biological behaviors, including survival, proliferation and differentiation. Niche signals including gap junction, neurotrophic factors and their downstream signals, ERK and JNK, were involved. Efforts for maintaining the direct cell-cell connection is worth making and it would provide a more favorable niche for NSCs/NPCs survival, proliferation and neuronal differentiation.

## Author Contributions

QJ, HL and YL conceived and designed the experiments; manuscript preparation. QJ performed the experiments. XL and JA contributed reagents/materials/analysis tools. QJ and ZZ analyzed the data. JT, XC and PZ helped perform the analysis with constructive discussions.

## Conflict of Interest Statement

The authors declare that the research was conducted in the absence of any commercial or financial relationships that could be construed as a potential conflict of interest.
