# Supplementary material for: Cell-Cell Connection Enhances Proliferation and Neuronal Differentiation of Rat Embryonic Neural Stem/Progenitor Cells
Source: Front Cell Neurosci. 2017 Jul 21;11:200. doi: 10.3389/fncel.2017.00200 (PMC5519523; doi:10.3389/fncel.2017.00200)
Supplement: Supplementary file 1 [file Data_Sheet_1.docx]

Supplementary Material

**Cell-cell connection enhances proliferation and neuronal differentiation of rat embryonic neural stem/progenitor cells**

Qian Jiao, Xingxing Li, Jing An, Zhichao Zhang, Xinlin Chen, Jing Tan, Pengbo Zhang, Haixia Lu* and Yong Liu*

# Supplementary figure 1: Viability of NSCs/NPCs after the treated with MAP kinase inhibitors


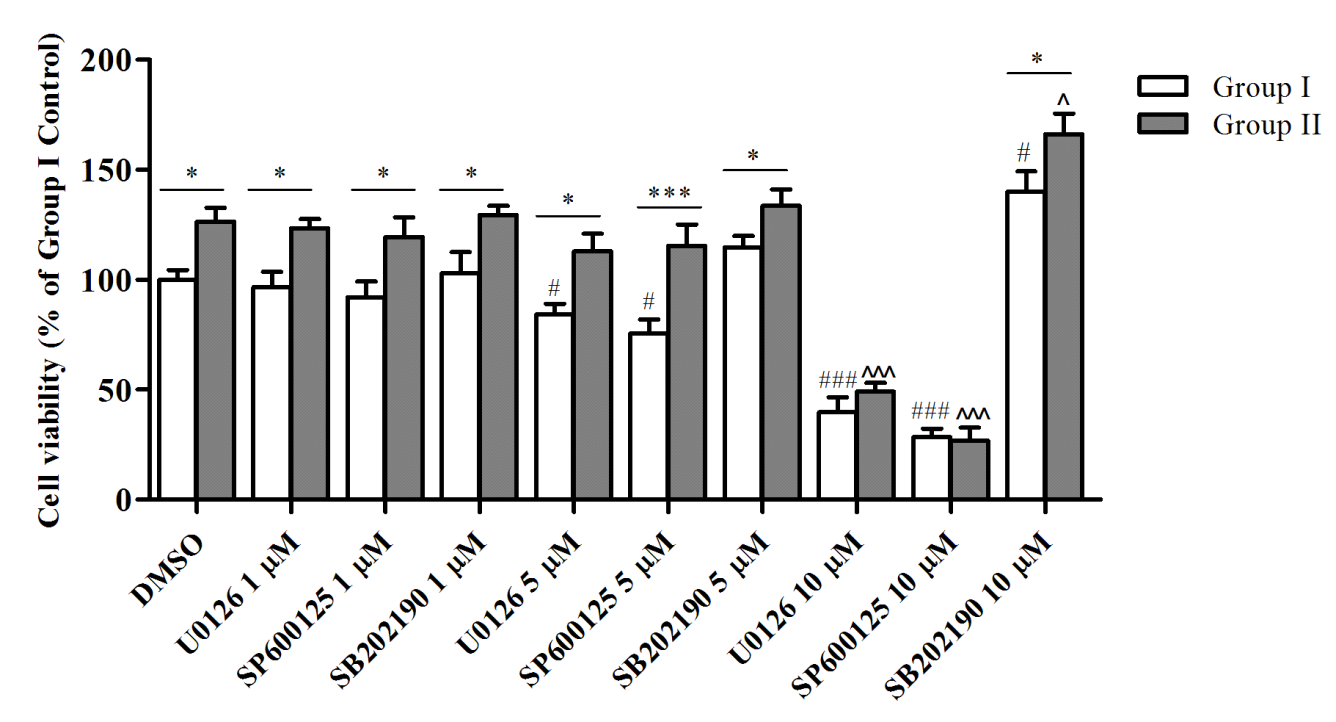


Viability of NSCs/NPCs after the treatment of MAP kinase inhibitors

**Legend:** Treatment with lower concentration of inhibitors showed no significant reduction of NSCs/NPCs viability compared with control (DMSO) in Group II, but in Group I, cell viability was significantly inhibited after treated with 5 μM of U0126 and SP600125 (# *P*< 0.05). On the other hand, after treated with 10 μM of U0126 and SP600125, viability of NSCs/NPCs in both groups dramatically declined (### *P*< 0.001 and ^^^ *P*< 0.001) and the difference between these two groups was disappeared. No reduction was observed after treatment with SB202190. All data were obtained from three independent experiments and 5 replicates for every experiment. The values are mean ± SD, analyzed via Student’s T test and one-way ANOVA. **P*< 0.05 and ****P*< 0.001 (via Student’s T test); #*P*< 0.05 and ###*P*< 0.001 represent Group I vs control (DMSO) (via one-way ANOVA); ^*P*< 0.05 and ^^^*P*< 0.001 represent Group II vs control (DMSO) (via one-way ANOVA).

# Supplementary figure 2: Inhibition of ERK and JNK phosphorylation


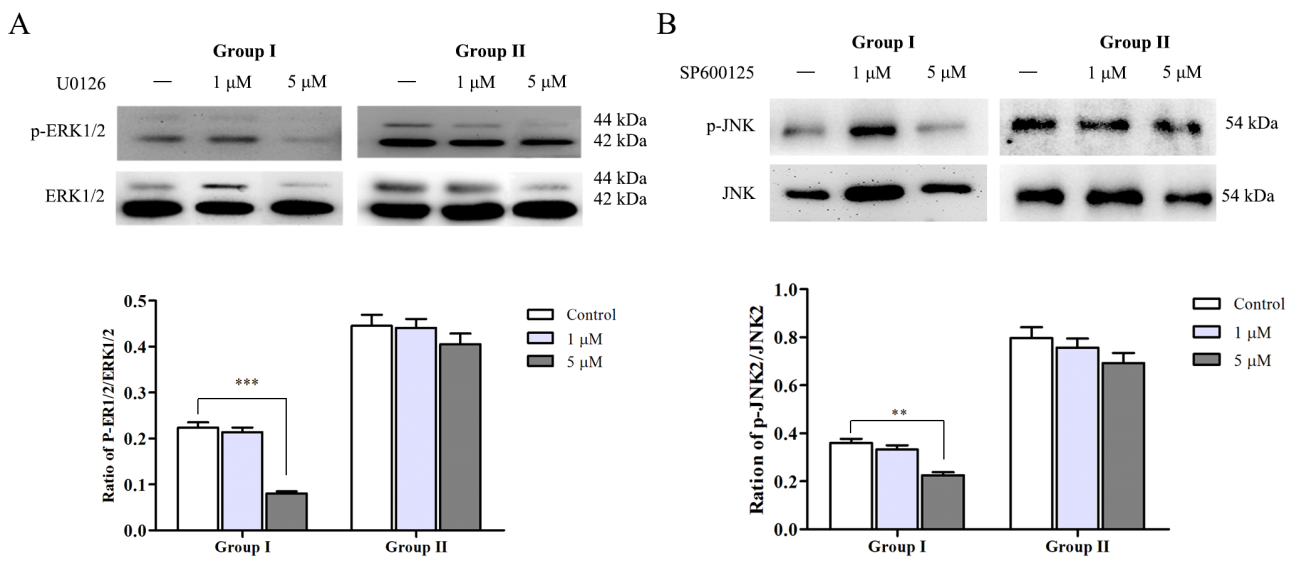


Inhibition of ERK and JNK phosphorylation

**Legend**: No significant inhibition of ERK (A) and JNK (B) phosphorylation was detected, in Group II, after the treatment with inhibitors either in 1 μM or 5 μM. Nevertheless, in Group I, significant inhibition of ERK (A) and JNK (B) phosphorylation was observed after treated with 5 μM of U0126 and SP600125. The values are mean ± SD, analyzed via one-way ANOVA. ***P*< 0.01 and ****P*< 0.001.

**Supplementary figure 3: Differentiation of NSCs/NPCs after treated with 5 μM of U0126**


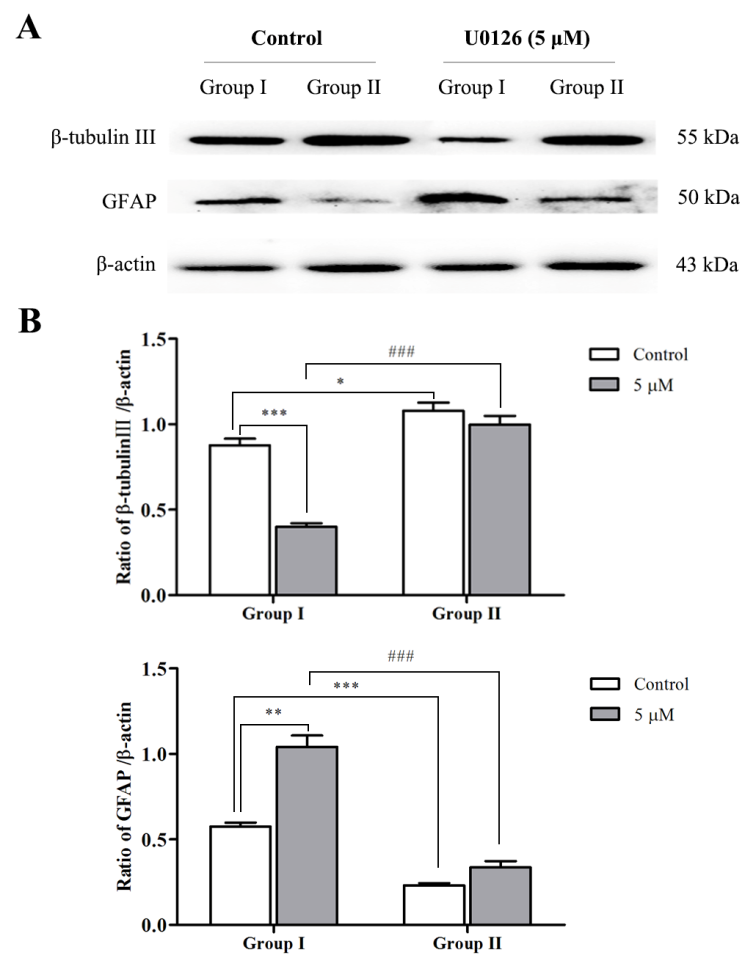


Differentiation of NSCs/NPCs after treated with 5 μM of U0126

**Legend**: In comparison with control, astrocytic differentiation of NSCs/NPCs was enhanced and the neuronal differentiation reduced significantly after treatment with 5 μM of U0126 in Group I while in Group II, no difference was observed. Notably, differences regarding astrocytic and neuronal differentiation between two groups were maintained. A: Western blot analysis of β-tubulin III and GFAP. B: Quantification of protein levels. The values are mean ± SD, analyzed via Student’s T test. **P*< 0.05, ***P*< 0.01 and ****P*< 0.001; ###*P*< 0.001.
